# Acoustic Source Localization Based on the Two-Level Data Aggregation Technology in a Wireless Sensor Network

**DOI:** 10.3390/s25072247

**Published:** 2025-04-02

**Authors:** Yuwu Feng, Guohua Hu, Lei Hong

**Affiliations:** College of Advanced Manufacturing Engineering, Hefei University, Hefei 230000, China; huguohua@hfuu.edu.cn (G.H.); hongleio@hfuu.edu.cn (L.H.)

**Keywords:** wireless sensor network, acoustic source localization, mixed noise model, data redundancy, data aggregation, energy consumption

## Abstract

The inherent energy constraints of sensor nodes render energy efficiency optimization a critical challenge in wireless sensor network deployments. This study presents an innovative acoustic source localization framework incorporating a two-level data aggregation technology, specifically designed to minimize energy expenditure while prolonging network lifetime. A mixed noise model is proposed to describe the characteristics of abnormal noise in real environments. Subsequently, the novel two-level data aggregation technology is proposed. The first level is implemented at individual sensors, where a large number of similar measurements may be collected. The second level data aggregation technology is performed at the cluster head nodes to eliminate the data redundancy between different sensor nodes. After the novel two-level data aggregation, most of the redundant data are eliminated and a significant amount of energy is saved. Then, a nonlinear iterative weighted least squares algorithm is applied to complete the final acoustic source location estimation based on the real remaining sensor measurements. Finally, through extensive simulation experiments, it was verified that the two-level data aggregation technology reduced energy consumption by at least 51% and 43%, respectively, and that the RMSE is less than 0.96.

## 1. Introduction

Wireless sensor networks are one of the emerging research hotspots in the field of information and have a wide range of applications in various fields, including environmental monitoring, smart homes, industrial automation, military applications and source localization [1]. Among these, acoustic source localization technology plays a very important role in the application of wireless sensor networks. The pseudo-linear formulation was employed to develop positioning algorithms for angle-of-arrival (AOA) localization, enabling target positioning through angular estimation of incoming signals [2]. The localization method based on AOA is suitable for narrowband signals, and nodes need to be arranged in an array with the same spacing. In [3], it developed an objective function based on time-difference-of-arrival (TDOA) measurements, integrating node coordinates to achieve acoustic source localization through spatiotemporal signal analysis. The localization method based on TDOA requires precise time synchronization, which can be challenging for wireless sensor networks. Li D. proposed a localization algorithm based on the received signal strength (RSS) and established a model of the relationship between energy attenuation and distance when sound signals propagated in the air [4]. Considering the computational complexity and conditional constraints, it is an appropriate choice for acoustic source localization in a wireless sensor network.

Due to the fact that sensor nodes mainly rely on irreplaceable batteries in wireless sensor networks, it has become one of the biggest challenges to reduce energy consumption and extend network lifespan in wireless sensor networks. Recently, some distributed methods were presented to conserve energy. A general class of distributed algorithms for wireless sensor networks was proposed to complete source positioning or detection [5,6]. Blatt D. presented a distributed version of the projection-onto-convex-sets (POCSs) method to addresses the problem of locating an acoustic source [7]. In order to minimize power consumption, for a POCS algorithm, each sensor node executed its primary computational workload locally using onboard data, thereby eliminating the requirement for centralized aggregation of the complete dataset at a processing hub. Zhang Y. proposed a unified distributed alternating projection algorithm to solve the source localization for wireless sensor networks based on RSS [8]. The unified distributed alternating projection framework specifically enforced localized neighborhood interactions among sensor nodes, operating under dynamic jointly connected communication graphs that preserved topological connectivity over time. These proposed distributed algorithms reduce the transmission of a certain amount of communication data by optimizing the distributed network structure, in order to reduce energy consumption. Liu Y. proposed a distributed, robust source location estimation method using acoustic signatures in a wireless sensor network, which applied the fully distributed network structure and reduced mass data long-range transmission [9]. In the fully distributed network structure, each sensor node is regarded as a cluster head node, receiving data from other sensor nodes within a single hop range and completing preliminary position estimation, rather than the conventional clustering structure. To some extent, these locally distributed or fully distributed structures can reduce a certain amount of data transmission, but this does not reduce the collection of data from the source node, and the effect is not ideal.

However, in order to minimize energy consumption, it is necessary to reduce the amount of data transmitted from the beginning of data collection. In wireless sensor networks, a large number of sensor nodes continuously collect on-site data according to a set cycle, which has the following characteristics. One is that the data collected by a single sensor node in a short period of time have extremely high similarity, and the other is that the data collected by neighboring sensor nodes at similar times also have a high degree of similarity. In a word, the raw data collected by sensor nodes have a high degree of redundancy. Data aggregation technology can effectively reduce data redundancy to achieve the goal of reducing energy consumption and extend the service life of the network [10].

Data aggregation technology has always been one of the hot research areas that has attracted the attention of many researchers in wireless sensor networks. In recent years, researchers have also proposed many unique data aggregation algorithms. Intanagonwiwat explored the directed diffusion paradigm for such coordination to achieve the data aggregation [11]. It embodied a data-centric paradigm where communication exclusively coordinates information exchange through named data entities. Directed diffusion needed to be completed by selecting empirically good paths and by caching and processing data in-network and needed complex computation. In [12], Min D. introduced the data aggregation that was based on both tree and flow routing protocols during the process of data collection to reduce redundant transmissions instead of an efficient cluster topology control structure. The tree-based scheme was presented to balance energy consumption before any node’s failure due to total residual energy depletion and the flow-based scheme was spread to increase network lifetime. In [13], Yin applied a prediction model for data aggregation based on the adaptive step size least mean square (ASS-LMS) to minimize the communication cost. The ASS-LMS algorithm reduced energy consumption in terms of computation and data accuracy, rather than reducing the transmission of raw data. Abdalkafor analyzed the impact of a data aggregation strategy on the performance of wireless sensor networks and provided some insights for researchers by understanding how aggregation technology affected the performance of the wireless sensor networks in terms of energy efficiency [14].

In this work, a novel two-level data aggregation technology is proposed to reduce energy consumption, where the cluster-based network topology structure is used in the wireless sensor network. The entire wireless sensor network is divided into several small cluster structures and every small cluster has a cluster-head, which is used to manage each node within this cluster. Firstly, due to the raw data collected by sensor nodes with a high degree of redundancy, the first level data aggregation is performed at each sensor node. Then, the second level data aggregation is performed at the cluster head node to eliminate the data redundancy between different sensor nodes. After the novel two-level data aggregation technology, the redundancy of data is greatly reduced and then the cluster head nodes transfer the remaining valid data to the sink node to complete the acoustic source localization.

In order to complete the estimation of acoustic source location based on the RSS, many localization methods were proposed in previous studies. In [15], Shi Q. proposed a normalized incremental sub-gradient algorithm to perform the source localization. Although its computational complexity is relatively low, its positioning accuracy is not high. In [16], an algebraic closed-form solution for the acoustic source localization problem using energy measurements was proposed and the proposed solution reached the Cramer–Rao lower bound accuracy for Gaussian noise. It has high positioning accuracy, but high computational complexity. In [17], a novel quadratic-term elimination method was presented to improve the localization accuracy. In [18], Meng C. transformed the traditionally nonlinear and non-convex problem into convex optimization problems to complete acoustic source localization estimation. These two algorithms were sensitive to the initial value setting and were prone to getting stuck in local optima. Nevertheless, these developed approaches were all based on a Gaussian noise model and the positioning accuracy was not that high.

Regarding the complexity and variability of environmental noise, such as gusts, animal movements or adverse weather conditions, the conventional Gaussian noise model can no longer meet the computational needs. So, a mixed noise model is proposed in this paper and it is very suitable for the noise environment requirements in wireless sensor networks. Moreover, with this model, a low-power acoustic source localization algorithm based on the two-level data (LASL-TD) aggregation technology is proposed so that the redundancy of large amounts of similar measurements at the sensor node and between sensor nodes is eliminated. Based on the remaining valid data and the mixed noise model, the acoustic source location is estimated using a nonlinear iterative weighted least squares algorithm which is appropriate to solve a nonlinear optimization problem.

The contributions of this paper mainly include two aspects:

(1) A mixed noise model is proposed to describe the characteristics of abnormal noise in real environments and verify the effectiveness of the model with experimental data;
(2) Novel two-level data aggregation technology is developed to maximize the elimination of data redundancy, thereby reducing energy consumption and extending the lifecycle of wireless sensor networks.

The remainder of the paper is organized as follows. In Section 2, three types of model structures are provided. In Section 3, the two-level data aggregation technology is given. Section 4 describes the acoustic source localization algorithm. Simulation results are presented in Section 5. Conclusions are drawn in Section 6.

## 2. Models

### 2.1. Network Topology Structure

In this paper, a cluster-based network topology structure is applied where the entire wireless sensor network is divided into several clusters [19]. Each cluster contains several sensor nodes and each cluster has a cluster head node. Sensor nodes autonomously execute scheduled data acquisition in accordance with prescribed sampling regimes, while simultaneously performing data conversion and processing. The cluster head node is responsible for collecting perceptual data from member nodes within the cluster and performing data aggregation processing to reduce data redundancy, and improve network efficiency and data quality. The data transmission between the cluster head node and the member nodes is a single hop communication.

**Figure 1:**
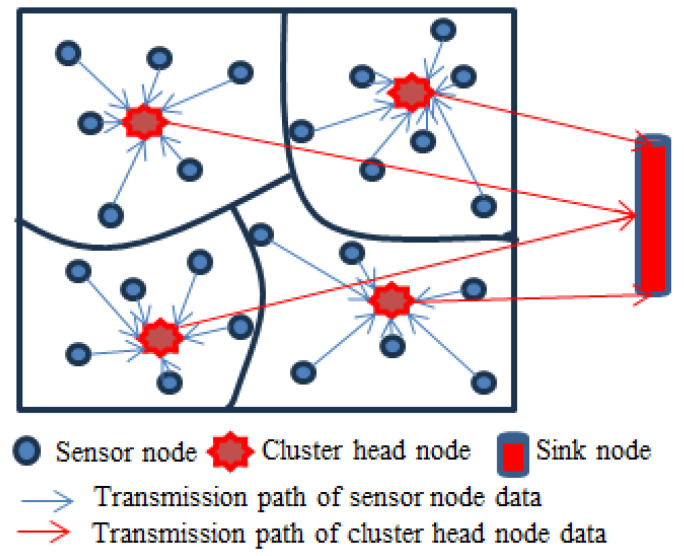
A cluster-based network topology structure.

In Figure 1, a cluster-based network topology structure is presented where data transmission in a cluster or between the cluster head node and sink node is based on the single hop communication.

### 2.2. Signal Attenuation Model

Assuming randomly deploying N sensor nodes within the monitoring area of a wireless sensor network, their positions are known and are denoted by $\left\{l_{n},\;\;1\leq n\leq N\right\}$. At the kth time, a source at location $\tau_{sk}$ is emitting an acoustic signal with the constant energy S (measured at 1 m away from the source). The energy value expression of the signal detected by the nth sensor node in the wireless sensor network at time k is expressed by [20]:

(1) $$y_{n}(k)=f(\theta (k))+e_{n}$$

(2) $$f(\theta (k))=\frac{g_{n}S}{{\left\|l_{n}-\tau_{sk}\right\|}^{2}}=\frac{g_{n}S}{d_{n}^{2}(k)}$$

where $g_{n}$ is the gain of the nth sensor node and $d_{n}(k)=\left\|l_{n}-\tau_{sk}\right\|$ is the Euclidean distance between the nth sensor node and source. $\theta (k)=\left\{S,\tau_{sk}\right\}$ is the unknown variable and the goal of acoustic source localization is to estimate these variations. Under normal circumstances, $e_{n}\;\sim \;N(\mu_{n},\sigma_{n}^{2})$ is a generalized steady-state Gaussian noise whose mean is $\mu_{n}$ and standard deviation is $\sigma_{n}$ and their values are estimated from sampled data. For the convenience of representation in the following paper, the time variable k will be omitted.

### 2.3. The Mixed Noise Model

Due to the complexity of environmental noise, such as sudden roars, occasional strong winds, occasional car honking and so on, the generalized Gaussian distribution noise model cannot accurately describe the characteristics of such noise. In recent years, some temporary remedies have been proposed to address this problem [21,22]. These methods start with negative impacts rather than actively mitigating the damage. This article proposes a mixed noise model to describe the presence of these anomalous noises:

(3) E=(1−ς)G+ςV

where ς is the prior experience factor, G is the Gaussian probability distribution and is the same as in Equation (1). V is an asymmetric probability distribution with much greater dispersion than the Gaussian probability distribution. In practical experiments, this article assumes that V is a uniformly distributed function, expressed as follows:

(4) $$V(0,\Gamma )=\left\{\begin{array}{c} 1/(\Gamma +1),0<e_{n}<\Gamma \\ 0,\;\;\;\;\;\;\;\;others \end{array}\right.$$

where Γ can dynamically take values based on sampled data, such as Γ=255 for 8-bit unsigned integers.

Similar to the Cauchy distribution, the mixed noise model belongs to a single-sided trailing tail distribution. When abnormal noise exists, the mixed noise model is very suitable for describing the abnormal noise that appears in a wireless sensor network.

In order to verify the effectiveness of the mixed noise model, this article conducted experiments in two different environments without acoustic source signals. One was in a specific laboratory without any abnormal external interference, and the other was on a normal school lawn. Acoustic sound noise is collected using a specific sensor in the experiment. The data type is an 8-bit unsigned integer with a frequency of 512 Hz. A total of 307,200 data are collected within 10 min and one acoustic noise reading is calculated for each 200-sample of data.

**Figure 2:**
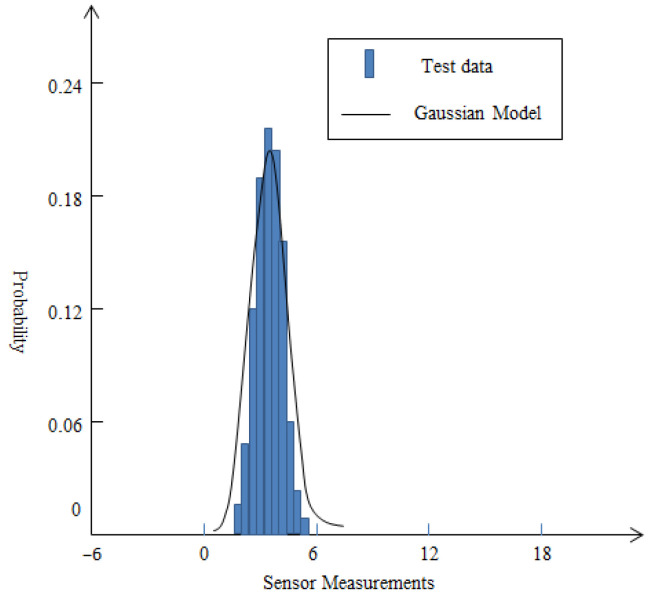
Distribution of measurement data in a specific laboratory.

**Figure 3:**
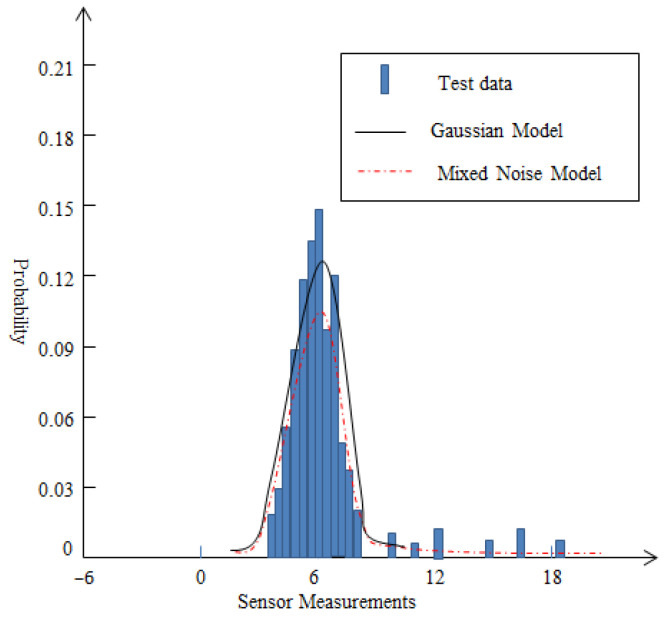
Distribution of measurement data on a normal school lawn.

Based on the measurement data of sensor nodes in two different environments, histograms are drawn as shown in Figure 2 and Figure 3. For Figure 2, according to the measurement data, the mean value is 3.98 and standard deviation value is 1.67 approximately. From Figure 2, it can be seen that the energy measurement values of the background acoustic noise are very close to the conventional Gaussian distribution. For Figure 3, in a complex school lawn environment, the mean and standard deviation of the measurement data are 6.46 and 2.39. From Figure 3, it can be seen that the background noise of the sound is disturbed by external loud noise forming a single-sided trailing distribution. For this special case, the conventional Gaussian distribution may not be suitable for describing its unique characteristics. On the contrary, the mixed noise model proposed in this article may be more suitable. When the value of the prior experience factor is 0.28, the form of the mixed noise model can be expressed as:

(5) $$\left(1-0.28)N(6.46,2.39^{2})+0.28(0,255\right)$$

It is noted that non-Gaussian noise usually refers to the random noise whose statistical characteristics deviate from Gaussian distribution, and its characteristics are usually manifested as spikes, heavy tails, asymmetry, or suddenness. From Figure 3, it can be seen that the presented mixed noise in this paper exhibits a single-sided trailing tail. In fact, it also belongs to a type of non-Gaussian noise, which exhibits a heavy tailed distribution due to the interference of sudden pulse noise. Compared to common non-Gaussian noise models, such as the Middleton Class A noise model, the mixed noise model presented in this paper has simpler calculations and simpler characterization of environmental characteristics, such as a sudden pulse-like noise interference.

## 3. Two-Level Data Aggregation Technology

**Figure 4:**
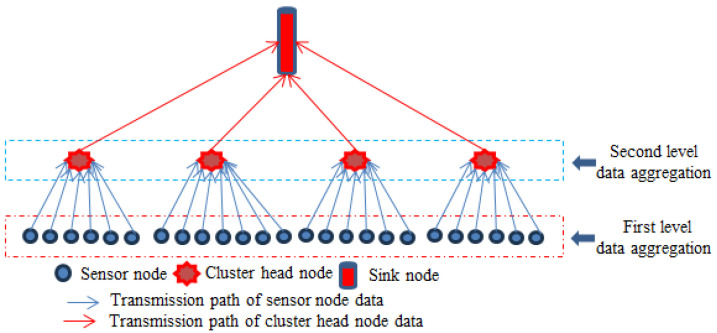
The two-level data aggregation structure.

In wireless sensor networks, a large number of sensor nodes continuously collect detection data according to a set cycle. If the position of the acoustic source remains unchanged or changes very slowly in a short period of time, the collected energy data have the following characteristics: the data collected multiple times by a single sensor node in a short period of time have high similarity, and the data collected by adjacent sensor nodes at similar times also have high similarity. Therefore, the data collected by sensor nodes have a high degree of redundancy. In order to reduce energy consumption and extend the service life of wireless sensor networks, it is necessary to perform the two-level data aggregation (TL-DAT) to reduce the transmission of data volume, as shown in Figure 4.

### 3.1. First Level Data Aggregation Technology (FL-DAT)

**Figure 5:**
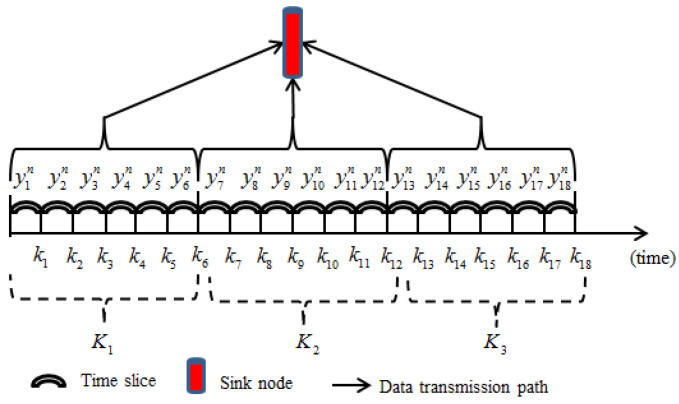
Data collected by sensor node.

In wireless sensor networks, a period of time K is divided into several equal time periods $K=\left[K_{1},K_{2},K_{3}\cdots \right]$, and each time period is subdivided into q different moments, meaning that each sensor node collects data q times in an evenly distributed time period. For example, when the value of q is 6, the expression can be written as following: $K_{1}=[k_{1},k_{2},\cdots ,k_{6}]$, $K_{2}=[k_{7},k_{8},\cdots ,k_{12}]$, $K_{3}=[k_{12},k_{13},\cdots ,k_{18}]$. If each time period $K_{1},K_{2},K_{3}\cdots$ is relatively short, then the data collected by the nth sensor node at six different times in each time period have a high similarity as shown in Figure 5.

Assuming that the nth sensor node collects a large amount of data during a certain time period, the data vector can be represented as

(6) $$Y_{n}=[y_{n}^{1},y_{n}^{2},\cdots ,y_{n}^{m_{n}}]$$

When the position of the acoustic source remains unchanged or changes very slowly in a short period of time, the data collected by the nth sensor node in a short period of time may have high redundancy, including mass similar measurement data. In order to eliminate the redundancy of data vector $Y_{n}$, the nth sensor node needs to search for similar data in vector $Y_{n}$. For the nth sensor node, define the similarity function between two measurements $y_{n}^{i}$ and $y_{n}^{j}$ for the first level data aggregation as follows:

(7) $$sf(y_{n}^{i},y_{n}^{j})=\left\{\begin{array}{c} 1,\left|y_{n}^{i}-y_{n}^{j}\right|\leq \varepsilon \\ 0,\left|y_{n}^{i}-y_{n}^{j}\right|>\varepsilon \end{array}\right.$$

where $y_{n}^{i}\in Y_{n},y_{n}^{j}\in Y_{n}$ and ε is the threshold, and the threshold value can be determined according to the actual environmental conditions. The selection of the parameter ε threshold generally requires comprehensive consideration of environmental factors, practical applications, and positioning accuracy. For example, in cases where environmental factors are complex, the threshold selection should not be too large, and a reasonable value should be less than 0.1. Normally, the selection of this threshold needs to be based on experience and practical application scenarios. When the function $sf(y_{n}^{i},y_{n}^{j})$ value is 1, the collection quantity $y_{n}^{i}$ and $y_{n}^{j}$ is similar. In order to maintain the integrity of measurement information, the weight values of measurement information are used to represent similar measurement data in the measurement data vector, denoted as $w(y_{n}^{i})$.

Based on the above statement, for the nth sensor node, we describe the general process of the first level data aggregation and summarize it as Algorithm 1.
**Algorithm 1.** The Process of first level data aggregation**1.**    Require: already measurement vector $Y_{n}$ **2**.              A new measurement $y_{n}^{j}$ **3.**    Goal: search for similarities in $Y_{n}$ **4.**    For each measurement $y_{n}^{i}\in Y_{n}$ do **5.**    if $sf(y_{n}^{i},y_{n}^{j})=1$ then **6.**           $w(y_{n}^{i})=w(y_{n}^{i})+1$ **7.**         Delete $y_{n}^{j}$ **8.**     else **9.**         append $y_{n}^{j}$ to $Y_{n}$ **10.**  $w(y_{n}^{j})=1$ **11.**    end if **12.**    end for

After the first level data aggregation technology, the measurement data vector $Y_{n}=[y_{n}^{1},y_{n}^{2},\cdots ,y_{n}^{m_{n}}]$ will be transformed into a weighted data vector Yn′=[(yn1,w(yn1)),⋯,(ynmn′,w(ynmn′))] with no redundancy. Then, the sensor nodes transmit the data to their respective cluster head nodes, which will perform the second level data aggregation.

### 3.2. Second Level Data Aggregation Technology (SL-DAT)

The cluster head node will receive the data which are sent from other sensor nodes in the cluster. Data redundancy still exists between different sensor nodes in the cluster. In order to further save the energy consumption, the cluster head node still needs to eliminate redundancy of the data received from different sensor nodes. It is a very important method for searching for similarity between different data vectors using distance functions and researchers have also proposed some distance functions recently [23]. In this chapter, a new second level data aggregation method based on Euclidean distance is presented to eliminate data redundancy of cluster head nodes.

For two sensor nodes n and η belonging to the same cluster, the data that transmitted to the same cluster head node are defined as Yn′=[(yn1,w(yn1)),⋯,(ynmn′,w(ynmn′))] and Yη′=[(yη1,w(yη1)),⋯,(yηmη′,w(yηmη′))], respectively. On this basis, the following similarity function model based on Euclidean distance is defined as:

(8) $$E_{d}f(Y_{n}^{\prime },Y_{\eta }^{\prime })=\left\{\begin{array}{c} 1,\sqrt{{\left(Y_{n}^{\prime }-Y_{\eta }^{\prime }\right)}^{2}}\leq \varphi \\ 0,\sqrt{{\left(Y_{n}^{\prime }-Y_{\eta }^{\prime }\right)}^{2}}>\varphi \end{array}\right.$$

where φ is the threshold, and the threshold value can be determined according to the actual environmental conditions. When the function $E_{d}f(Y_{n}^{\prime },Y_{\eta }^{\prime })$ value is 1, the collection quantity $Y_{n}^{\prime }$ and $Y_{\eta }^{\prime }$ is similar.

To calculate the Euclidean distance between two sensor nodes n and η with respect to the collected quantities $Y_{n}^{\prime }$ and $Y_{\eta }^{\prime }$, it is necessary to split the collected quantities into two parts: the intersection part and the dissimilarity part. Define the intersection part as:

(9) $$Y_{n-\eta }=Y_{n}^{\prime }\cap Y_{\eta }^{\prime }=\left\{y_{n}^{i}\in {Y^{\prime }}_{\eta },y_{\eta }^{j}\in {Y^{\prime }}_{n},sf(y_{n}^{i},y_{\eta }^{j})=1,w_{\cap }(y_{n}^{i},y_{\eta }^{j})=\min(w(y_{n}^{i}),w(y_{\eta }^{j}))\right\}$$

For the collection quantity $Y_{n}^{\prime }$, define the dissimilarity part as:

(10) $$Y_{n}^{\prime ⊄}=Y_{n}^{\prime }\Theta Y_{n-\eta }=Y_{n}^{\prime }\Theta (Y_{n}^{\prime }\cap Y_{\eta }^{\prime })$$

For the collection quantity $Y_{\eta }^{\prime }$, define the dissimilarity part as:

(11) $$Y_{\eta }^{\prime ⊄}=Y_{\eta }^{\prime }\Theta Y_{n-\eta }=Y_{\eta }^{\prime }\Theta (Y_{n}^{\prime }\cap Y_{\eta }^{\prime })$$

where Θ represents the operation of finding the distinct parts between two datasets and the specific operation method is as follows: for two measurement values $y_{n}^{i}$ and $y_{\eta }^{i}$, if $y_{n}^{i}\in Y_{n}^{\prime }$, $y_{\eta }^{j}\in Y_{n-\eta }$ and $sf(y_{n}^{i},y_{\eta }^{j})=1$, then the dissimilarity operation is $Y_{n}^{\prime ⊄}=Y_{n}^{\prime }\Theta Y_{n-\eta }=\left\{y_{n}^{i}\in {Y^{\prime }}_{n},w(y_{n}^{i})=w(y_{n}^{i})-w(y_{\eta }^{j})\right\}$.

For computational convenience, it is necessary to convert data vectors $Y_{n}^{\prime ⊄}$ and $Y_{\eta }^{\prime ⊄}$ into a dataset as follows:

(12) $$Y_{n}^{\prime ⊄}=\left[\underset{number:w(y_{n}^{1})}{\underset{︸}{y_{n}^{1},\cdots ,y_{n}^{1}}},\underset{number:w(y_{n}^{2})}{\underset{︸}{y_{n}^{2},\cdots ,y_{n}^{2}}},\cdots ,\underset{number:w(y_{n}^{m_{n}^{\prime }})}{\underset{︸}{y_{n}^{m_{n}^{\prime }},\cdots y_{n}^{m_{n}^{\prime }}}}\right]$$

(13) $$Y_{\eta }^{\prime ⊄}=\left[\underset{number:w(y_{\eta }^{1})}{\underset{︸}{y_{\eta }^{1},\cdots ,y_{\eta }^{1}}},\underset{number:w(y_{\eta }^{2})}{\underset{︸}{y_{\eta }^{2},\cdots ,y_{\eta }^{2}}},\cdots ,\underset{number:w(y_{\eta }^{m_{\eta }^{\prime }})}{\underset{︸}{y_{\eta }^{m_{\eta }^{\prime }},\cdots y_{\eta }^{m_{\eta }^{\prime }}}}\right]$$

For two sensor nodes n and η belonging to the same cluster, according to the dataset $Y_{n}^{\prime ⊄}$ and $Y_{\eta }^{\prime ⊄}$, the formula for calculating the Euclidean distance of the collected quantity $Y_{n}^{\prime }$ and $Y_{\eta }^{\prime }$ is as follows:

(14) $$\begin{array}{l} E_{d}(Y_{n}^{\prime },Y_{\eta }^{\prime })=\sqrt{{\left(Y_{n}^{\prime }-Y_{\eta }^{\prime }\right)}^{2}}\\ \;\;\;\;\;\;\;\;\;\;\;\;\;\;\;\;\;=\sqrt{{\left(Y_{n-\eta }+Y_{n}^{\prime ⊄}\right)}^{2}-{\left(Y_{n-\eta }+Y_{\eta }^{\prime ⊄}\right)}^{2}}\\ \;\;\;\;\;\;\;\;\;\;\;\;\;\;\;\;\;=\sqrt{{\left(Y_{n}^{\prime ⊄}-Y_{\eta }^{\prime ⊄}\right)}^{2}}\\ \;\;\;\;\;\;\;\;\;\;\;\;\;\;\;\;\;=\sqrt{\sum_{m=1}^{\left|Y_{n}^{\prime ⊄}\right|}{\left(y_{n}^{m}-y_{\eta }^{m}\right)}^{2}} \end{array}$$

where $y_{n}^{m}\in Y_{n}^{\prime ⊄},y_{\eta }^{m}\in Y_{\eta }^{\prime ⊄}$ and $\left|Y_{\eta }^{\prime ⊄}\right|$ is equal to the number of elements in $Y_{\eta }^{\prime ⊄}$.

According to Formula (14), when the function $E_{d}f(Y_{n}^{\prime },Y_{\eta }^{\prime })$ value is 1, the collection quantity $Y_{n}^{\prime }$ and $Y_{\eta }^{\prime }$ is similar, so the data redundancy exists. The cluster head node selects one for data transmission as needed, while discarding the other. After the second level data aggregation, the data that need to be transmitted are further reduced, saving more energy consumption.

## 4. Acoustic Source Localization Algorithm

After the two-level data aggregation technology, each cluster head node transmits the valid data to the sink node, which completes the final estimation of the acoustic source location. Assume that the set $\hat{Y}=[y_{1},y_{2},\cdots ,y_{\tilde{N}}]$ is used to represent all the data received by the sink node and $\tilde{N}$ represents the number of effective sensors. M-estimation is a conventional maximum likelihood function estimation method, whose core idea is to minimize the objective function, as proposed by Huber in the literature [24]. At the sink node, based on the energy data received from all cluster head nodes and the mixed Gaussian noise model, a nonlinear iterative weighted least squares algorithm (NIWLSA) is presented to solve the M-estimation and the estimation of unknown acoustic source variables can be obtained by minimizing the objective function:

(15) $$l(\tilde{\theta })=\sum_{n=1}^{\hat{N}}\rho (\frac{r_{n}(\theta )}{{\hat{\sigma }}_{n}})$$

where $r_{n}(\theta )=y_{n}-f(\theta )-{\hat{\mu }}_{n}$ and ${\hat{\mu }}_{n},{\hat{\sigma }}_{n}$ represents the mean and standard deviation of the mixed Gaussian noise, respectively. The selection of objective function ρ(⋅) should consider its stability and efficiency. In this paper, the Bi-square function [25] is used to provide a balanced estimator and ρ(⋅) can be described as:

(16) $$\rho (x)=1-{\left(1-{\left(x/8\right)}^{2}\right)}^{3}$$

In this paper, for a nonlinear optimization problem with the objective function as described in (15), a nonlinear iterative weighted least squares algorithm is applied. Assuming $\nabla_{\theta }l(\tilde{\theta })=0$, we can obtain:

(17) $$\nabla_{\theta }l(\tilde{\theta })=\sum_{n=1}^{\hat{N}}\nabla_{\theta }l(\tilde{\theta })=\sum_{n=1}^{\hat{N}}\vartheta (\frac{r_{n}(\theta )}{{\hat{\sigma }}_{n}})\nabla_{\theta }(\frac{r_{n}(\theta )}{{\hat{\sigma }}_{n}})=0$$

where ϑ(x)=∂ρ(x)/∂x. Observe that when x→0,$\rho (x)\approx x^{2}$. Therefore, it is reasonable that when x→0,ζ(x)≈ϑ(x)/x and ζ(x) is approximately a constant. Furthermore, (17) can be transformed into the following form:

(18) $$\sum_{n=1}^{\hat{N}}\vartheta (\frac{r_{n}(\theta )}{{\hat{\sigma }}_{n}})\left[\frac{r_{n}(\theta )}{{\hat{\sigma }}_{n}}\right]\nabla_{\theta }f(\theta )=0$$

Then, the gradient of a weighted least square objective function can be approximately expressed in the following form:

(19) $$\sum_{n=1}^{\hat{N}}\vartheta (\frac{r_{n}(\theta )}{{\hat{\sigma }}_{n}})\left[\frac{r_{n}(\theta )}{{\hat{\sigma }}_{n}}\right]\nabla_{\theta }f(\theta )=\nabla_{\theta }\left\{\sum_{n=1}^{\hat{N}}\vartheta (\frac{r_{n}(\theta )}{{\hat{\sigma }}_{n}}){\left(r_{n}(\theta )\right)}^{2}\right\}$$

Ultimately, $\tilde{\theta }$ can be estimated using an approximate objective function:

(20) $$\underset{\theta }{\min}\sum_{n=1}^{\hat{N}}\vartheta (\frac{r_{n}(\theta )}{\sigma_{n}}){\left(y_{n}-f(\theta )-\mu_{n}\right)}^{2}$$

An iterative quasi-Newton algorithm can be used to solve the objective function in order to obtain an estimation of the acoustic source location [24]. The iterative quasi-Newton algorithm is an optimization algorithm used to solve unconstrained optimization problems. It approximates the Hessian matrix to avoid directly calculating the second derivative, thereby saving computational resources. The robustness of applying the quasi-Newton algorithm to estimate the position of the sound source is mainly reflected in the following three aspects: (a) this algorithm is relatively insensitive to the selection of the initial point of the sound source position, and can converge to the local optimal solution even at poor initial points; (b) this algorithm iteratively corrects the Hessian approximation and has a smoothing effect on small errors in gradient estimation, avoiding algorithm divergence caused by single gradient errors; (c) by gradually updating the Hessian approximation matrix, we can avoid directly calculating the potentially singular true Hessian matrix and reduce numerical instability. Therefore, it is very suitable to solve the position estimation problem by applying the quasi-Newton algorithm in wireless sensor networks.

## 5. Simulation Results

Within a 100 m×100 m two-dimensional monitoring area, there is a single acoustic source emitting the acoustic signal with a constant energy intensity; measured at 1 m away from the acoustic source, it is 5000. Assume that 100 sensor nodes are placed in the monitoring area. The sensor gain is identically 1. The environmental background noise is a mixed noise model, which includes a Gaussian noise model with a mean of 0.9 and a standard deviation of 0.4 and a non-Gaussian noise with a uniform distribution over [0, 255]. The sensor nodes collect q energy samples every 30 s, 300 s, 600 s and 900 s, respectively. The data transmission between the cluster head node and cluster member nodes is a single hop communication. The aggregation ratio is defined by the number of data measures sent to the total number of data measures generated. The threshold value of ε in first-level data aggregation is 0.04, 0.08, and 0.12, and the threshold value of φ is 0.3, 0.4, and 0.5.

### 5.1. Ratio of Two-Level Data Aggregation

**Figure 6:**
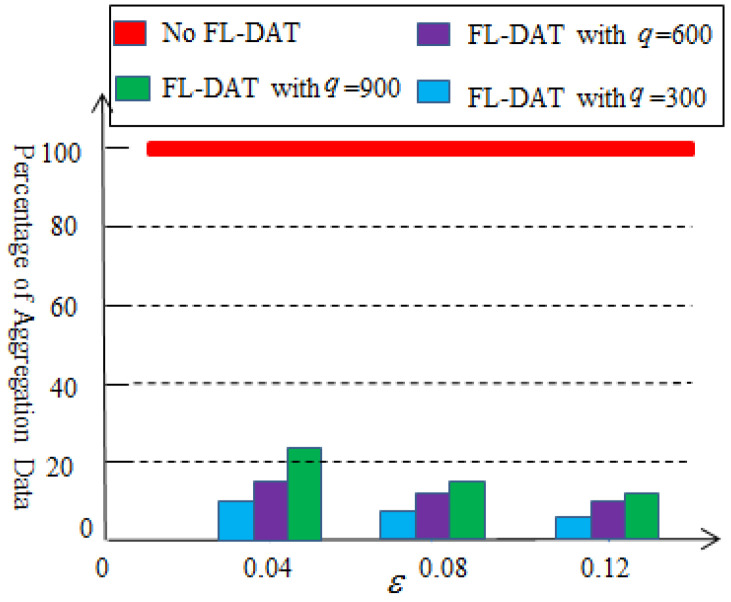
Percentage of the aggregation data based on the first-level aggregation.

Figure 6 illustrates the results of the ration of the first-level aggregation. The effectiveness of the first-level aggregation depends on the selection of threshold value and the amount of data collected. Without data aggregation, all data collected by sensor nodes will be transmitted to the cluster head node. However, after performing the first-level data aggregation, the amount of data that need to be transmitted will be significantly reduced, at least by 76%. Therefore, the first-level data aggregation technology can effectively reduce data redundancy. At the same time, it can also be observed that as the threshold value or collection volume increases, the effectiveness of the data aggregation improves.

**Figure 7:**
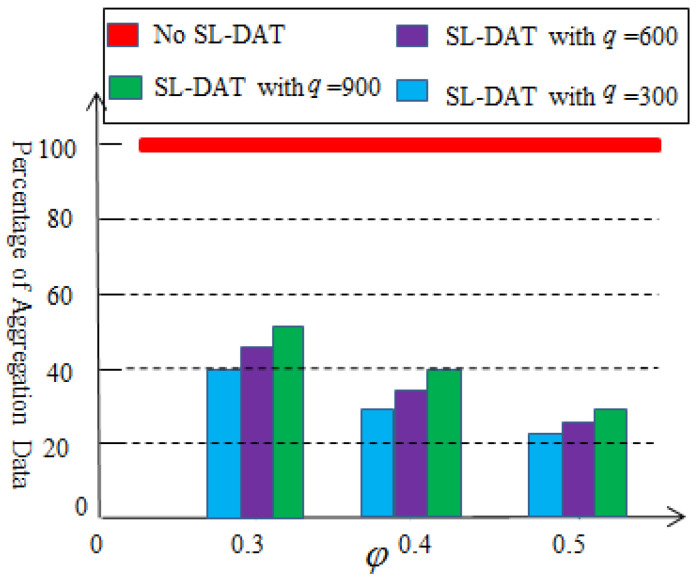
Percentage of the aggregation data based on the second-level aggregation.

Figure 7 illustrates the results of the ration of the second-level aggregation. The effectiveness of the second-level aggregation depends on the selection of the threshold value and the amount of data collected at the first-level aggregation. From Figure 7, after performing the second-level data aggregation, the amount of data that needs to be transmitted to the sink node will be further reduced, at least by 48%. Therefore, the second-level data aggregation technology can also effectively reduce the data redundancy. At the same time, it can also be observed that as the threshold value or collection volume increases, the effectiveness of data aggregation improves.

From Figure 6 and Figure 7, after the novel two-level data aggregation technology, the data that need to be transmitted to the sink node will be significantly reduced, at least by 87%. It can be seen that there is a large amount of data redundancy in the data collected by sensor nodes and the demand of the data aggregation technology for wireless sensor networks is necessary.

**Figure 8:**
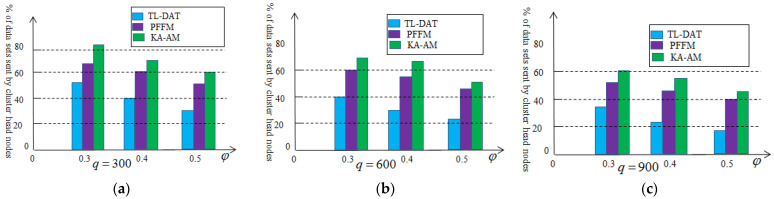
Percentage of the aggregation data based on the two-level aggregation: (**a**) q=300, (**b**) q=600, (**c**) q=900.

In order to compare with the two-level aggregation technology in this paper, a Prefix Frequency Filtering model (PFFM) algorithm [26] and K-means algorithm based on the Anova model (KA-AM) [27] are introduced. The PFFM algorithm is to identify near duplicate nodes that generate similar sets of collected data in periodic applications. The K-means algorithm was applied to identify nodes generating identical data sets. Figure 8 depicts the percentage of the aggregation data that need to be transmitted to the sink node to perform acoustic source location estimation. We vary φ and set q=300 in Figure 8a, q=600 in Figure 8b and q=900 in Figure 8c. As can be seen from Figure 8, the effectiveness of the two-level data aggregation technology is superior to the other two methods. The reason is that the two-level data aggregation technology makes it easier to search for data redundancy. As the amount of data collected increases, the effect will also be significantly enhanced.

### 5.2. Energy Consumption Analysis of the Two-Level Data Aggregation

For a sensor node, the energy consumed by transmitting p bit packets to the cluster head node at a distance of d can be expressed as the following equation [28]:

(21) $$E(p,d)=\left\{\begin{array}{c} E_{elec}\times p+\xi_{amp}\times p\times d^{2},d<d_{0}\\ E_{elec}\times p+\xi_{amp}\times p\times d^{4},d>d_{0} \end{array}\right.$$

where $E_{elec}$ represents the energy consumed by the sensor node during startup, $\xi_{amp}$ represents the amplifier energy and $d_{0}$ represents the single hop communications distance. In the clustering structure, the communication between the sensor node and cluster head node is a single hop communication. According to Equation (21), in clustered wireless sensor networks, the main factor that affects the energy consumption of the sensor node is the total amount of data transmitted. Therefore, in order to reduce the energy consumption of sensor nodes, the main purpose of the two-level data aggregation technology proposed in this paper is to minimize the amount of data that need to be transmitted as much as possible. In the simulation experiment, assuming that the communication energy parameters are set as $E_{elec}=50\;\mathrm{nJ}/\mathrm{bit}$ and $\xi_{amp}=10\;{\mathrm{pJ}/\mathrm{bit}/m}^{2}$.

**Figure 9:**
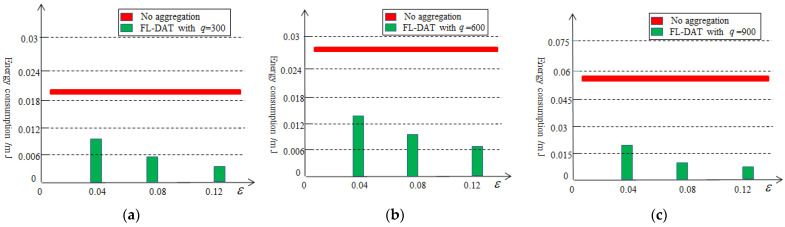
Energy consumption based on the first-level aggregation: (**a**) q=300, (**b**) q=600, (**c**) q=900.

Figure 9 illustrates the energy consumption of sensor nodes by varying q with or without the first-level data aggregation. From the graph, it can be seen that after the first-level data aggregation, the sensor nodes have reduced a significant amount of energy consumption, at least 51% approximately. When the q is 900 and ε is 0.12, the energy consumption has been reduced by over 84%. The reason is that the first-level data aggregation method eliminates a certain amount of data redundancy. As the threshold increases, the more the data redundancy is reduced and the more the energy consumption is reduced.

**Figure 10:**
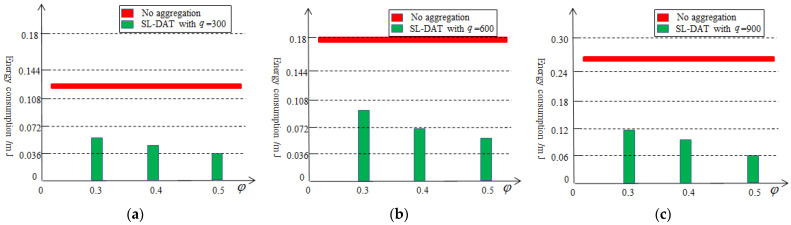
Energy consumption based on the second-level aggregation: (**a**) q=300, (**b**) q=600, (**c**) q=900.

Figure 10 illustrates the energy consumption of sensor nodes by varying q with or without the second-level data aggregation. From the graph, it can be seen that after the second-level data aggregation, the cluster head nodes have reduced a significant amount of energy consumption, at least 43% approximately. When the q is 900 and φ is 0.5, the energy consumption has been reduced by over 76%. As the threshold increases, the more the data redundancy is reduced and the more the energy consumption is reduced. Meanwhile, the greater the frequency of data collection is, the more energy is saved.

**Figure 11:**
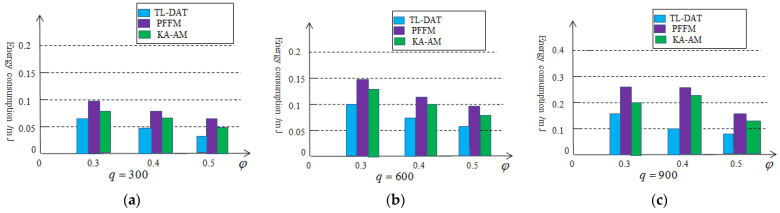
Energy consumption based on the two-level aggregation: (**a**) q=300, (**b**) q=600, (**c**) q=900.

Figure 11 illustrates the energy consumption of the cluster head nodes compared with PFFM algorithm and KA-AM algorithm. We vary φ and set q=300 in Figure 11a, q=600 in Figure 11b and q=900 in Figure 11c. Compared to the other two algorithms, the algorithm based on the two-level data aggregation saves more energy because the algorithm based on the second-level data aggregation requires fewer data to be transmitted from the cluster head nodes to the sink node.

**Figure 12:**
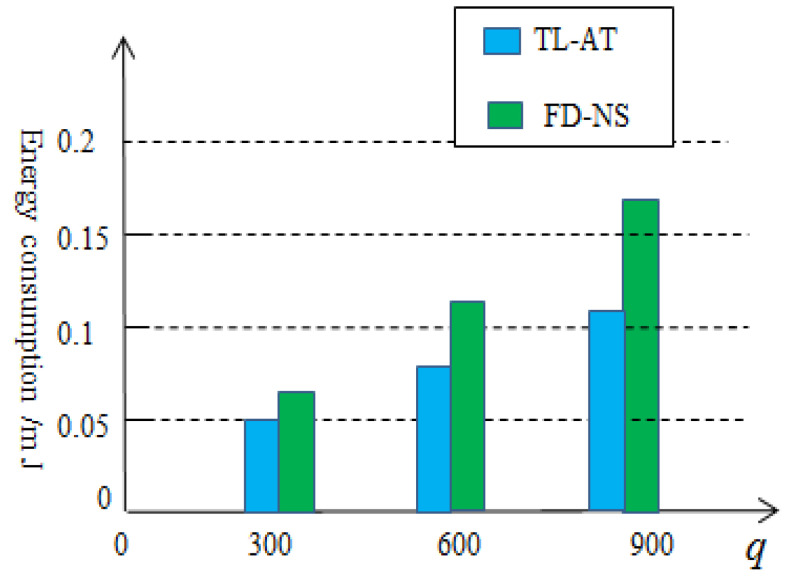
Comparison of energy-saving effects between two different methods.

In order to compare the energy-saving effect, it would be useful to compare the proposed two-level aggregation technology (TL-AT) with other energy-saving techniques for wireless sensor networks, such as a distributed network structure. In [29], a fully distributed network structure (FD-NS) was presented to reduce the energy consumption. In the fully distributed network structure, each sensor node is regarded as a cluster head node, receiving data from other sensor nodes within a single hop range and completing a preliminary position estimation, rather than the conventional clustering structure. Although it reduces the long-distance transmission of data, each sensor node will receive or send a large amount of collected data, which will also consume a lot of energy. In order to observe the effect, a simulation comparison is made between the fully distributed structure method and the two-level data aggregation method proposed in this paper, as shown in Figure 12. From Figure 12, it can be seen that the energy-saving method based on two-level data aggregation proposed in this paper is better than the fully distributed structure in the literature [29] and the effect will become more pronounced as the amount of data collected increases.

### 5.3. Localization Accuracy Analysis

**Figure 13:**
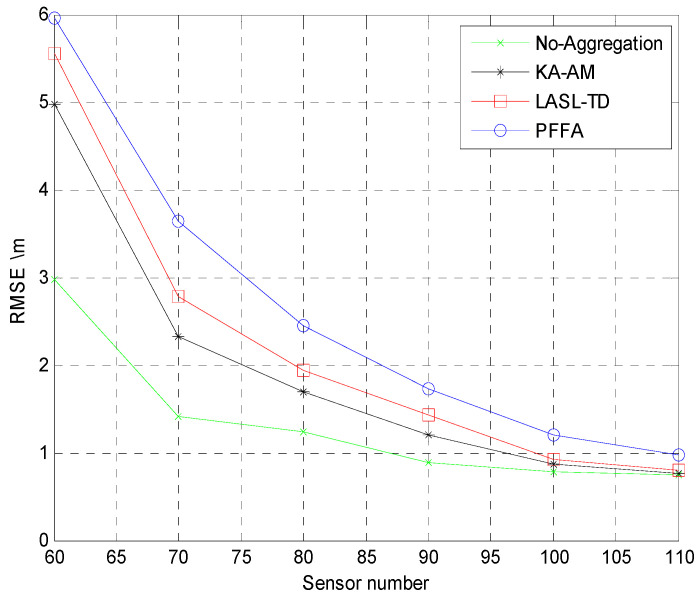
RMSE for a single source versus sensors.

For better comparison, the positioning algorithms all use the NIWLSA algorithm after data aggregation or no aggregation. The root mean square error (RMSE) for acoustic source localization using LASL-TD algorithm, Prefix Frequency Filtering algorithm (PFFA) algorithm, and K-means algorithm based on Anova model (KA-AM) are depicted in Figure 13. From Figure 13, we can see that when the number of sensors increases, the accuracy improves quickly at first and then smooths out. When the number of sensor nodes reaches 100, the RMSE of the LASL-TD proposed in this paper is less than 1. Meanwhile, the RMSE of the three algorithms is approaching the algorithm without using aggregation technology, but the LASL-TD proposed in this paper consumes the least energy. Therefore, the LASL-TD algorithm is suitable in wireless sensor networks with limited energy.

**Figure 14:**
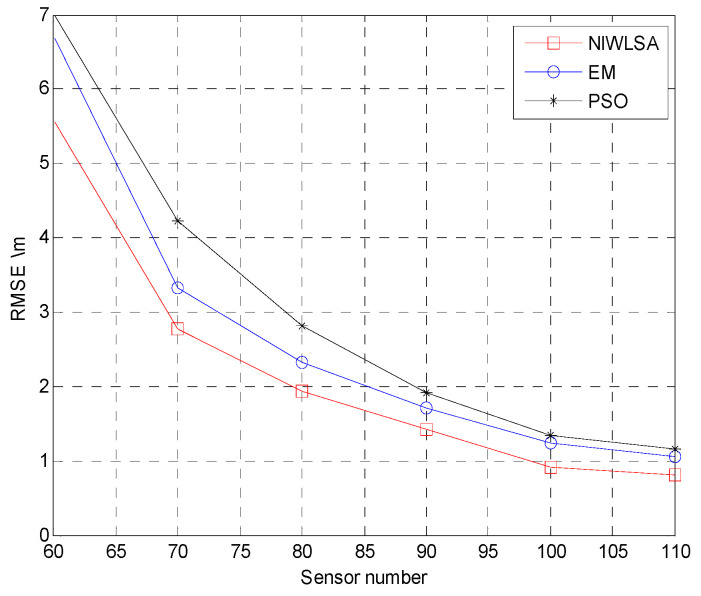
RMSE with different localization algorithms.

In order to compare the localization accuracy, different localization algorithms are applied with the same data aggregation technology proposed in this paper. In addition to NIWLSA in this paper, the other two localization algorithms are the EM algorithm [30] and the PSO algorithm [31]. From Figure 14, it can be seen that the localization accuracy of the algorithm proposed in this article is higher than the other two algorithms and has better adaptability to the mixed-noise model.

## 6. Conclusions

In this paper, to address the issue of limited energy consumption in wireless sensor networks, an acoustic source localization algorithm based on the novel two-level data aggregation is proposed to reduce the energy consumption and extend the lifecycle of wireless sensor networks. Firstly, a mixed-noise model is proposed to describe the characteristics of abnormal noise in real environments. Then, the novel two-level data aggregation technology is proposed. The first level is at the sensor node, where a large number of similar measurements may be collected. A similar function is proposed to eliminate the redundant data. Verified through the simulation experiments, after the first-level data aggregation, the sensor nodes reduced a significant amount of energy consumption, at least 51% approximately. The second-level data aggregation technology based on Euclidean distance is performed at the cluster head nodes to eliminate the data redundancy between different sensor nodes. Meanwhile, the cluster head nodes also reduced a significant amount of energy consumption, at least 43% approximately. After the novel two-level data aggregation, most of the redundant data are eliminated and a significant amount of energy is saved. Then, a nonlinear iterative weighted least squares algorithm is applied to complete the final acoustic source location estimation based on the real remaining sensor measurements. Finally, the effectiveness and accuracy of the proposed acoustic source localization based on the two-level data aggregation are clearly demonstrated through extensive simulation results.

This paper considers data collection and processing to reduce the energy consumption of sensor nodes. However, in wireless sensor networks, the sensor nodes are often waiting in idle mode for an interesting acoustic source to occur. Energy waste caused by this idle listening of sensor nodes is also a major source of energy waste in wireless sensor networks. To further improve the energy conservation, sleep–wake management scheme-based energy conservation mechanisms may constitute a pivotal research direction in future studies [32]. Furthermore, the synergistic combination leveraging the complementary strengths of these two approaches, the two-level data aggregation technology and sleep–wake management mechanism, may emerge as a promising research frontier in energy conservation studies.

## Data Availability

The raw data supporting the conclusions of this article will be made available by the authors on request.
